# Targeting Sepsis: Disease Tolerance, Immune Resilience, and Compartmentalized Immunity

**DOI:** 10.3390/biomedicines12112420

**Published:** 2024-10-22

**Authors:** Alexis Garduno, Ignacio Martín-Loeches

**Affiliations:** 1 Department of Intensive Care Medicine, Multidisciplinary Intensive Care Research Organization (MICRO), St. James’ Hospital, D08 NHY1 Dublin, Ireland; gardunoa@tcd.ie; 2 Hospital Clinic, Universitat de Barcelona, IDIBAPS, CIBERES, 08036 Barcelona, Spain

**Keywords:** sepsis, immune dysregulation, compartmentalized immune responses, immune resilience, epigenetics, organ-specific therapy, critical care

## Abstract

**Introduction:** Sepsis remains a major contributor to critical care mortality and morbidity worldwide. Despite advances in understanding its complex immunopathology, the compartmentalized nature of immune responses across different organs has yet to be fully translated into targeted therapies. This review explores the burden of sepsis on organ-specific immune dysregulation, immune resilience, and epigenetic reprogramming, emphasizing translational challenges and opportunities. **Methods:** We implemented a systematic literature search strategy, incorporating data from studies published between 2010 and 2024, to evaluate the role of molecular profiling techniques, including transcriptomics and epigenetic markers, in assessing the feasibility of targeted therapies. **Results:** Sepsis-induced immune dysregulation manifests differently in various organs, with lung, heart, liver, and kidney responses driven by unique local immune environments. Organ-specific biomarkers, such as the Spns2/S1P axis in lung macrophages, mitochondrial dysfunction in the heart, proenkephalin for early acute kidney injury (AKI), and adrenomedullin for predicting multi-organ failure, offer promising avenues for timely intervention. Furthermore, immune resilience, particularly through regulatory T-cell modulation and cytokine targeting (e.g., IL-18), is crucial for long-term recovery. Epigenetic mechanisms, including histone modification and trained immunity, present opportunities for reprogramming immune responses but require more precision to avoid unintended inflammatory sequelae. **Conclusions:** A deeper understanding of compartmentalized immune responses and the dynamic immune landscape in sepsis is critical for developing precision therapies. Real-time immune monitoring and organ-targeted interventions could revolutionize sepsis management, although significant barriers remain in clinical translation. Further research is required to establish biomarkers and treatment timing that optimize therapeutic efficacy while minimizing systemic risks.

## 1. Introduction

Sepsis is a multifaceted syndrome that represents one of the most significant challenges in critical care medicine today, causing over 11 million deaths annually and causing severe morbidity in survivors [1]. This complex life-threatening syndrome arises from a dysregulated immune response to infection, resulting in widespread inflammation and multi-organ dysfunction. The pathogenesis of sepsis can be understood through a biphasic immune response, comprising an initial hyperinflammatory phase, often described as a cytokine storm, followed by a profound and sustained immunosuppressive phase, which renders patients vulnerable to secondary infections and involves the reactivation of latent viruses and chronic critical illness (CCI) [1,2,3,4]. The shift from an overwhelming pro-inflammatory cytokine storm to immune paralysis marks one of the most confounding aspects of the disease, contributing to increased mortality, secondary infections, and long-term complications in survivors [5,6].

Sepsis is not a homogeneous condition, as immune responses vary widely across different organs and among individual patients. A significant focus has emerged on the compartmentalization of immune responses, where different organs such as the lungs, liver, kidneys, and heart exhibit distinct immune dysregulation. This compartmentalized immunity necessitates a tailored therapeutic approach, as organ-specific immune suppression or hyperactivation can drastically affect clinical outcomes [7,8].

Current diagnostics, which rely primarily on blood cultures, have limited sensitivity and often fail to detect non-bacterial pathogens, including fungi and viruses, which may be responsible for sepsis in a substantial subset of patients [9,10]. Moreover, blood cultures can take days to return results, by which time the window for effective early intervention may have passed. Beltrán-García et al. [11] highlighted the stark relationship between delayed intervention and increased mortality, emphasizing that for every hour treatment is delayed beyond the first six hours of infection, patient mortality increases by 7.8%. This underlines the urgency of developing rapid diagnostic tools that not only detect the pathogen but also provide insight into the immune status of the patient.

**Figure 1:**
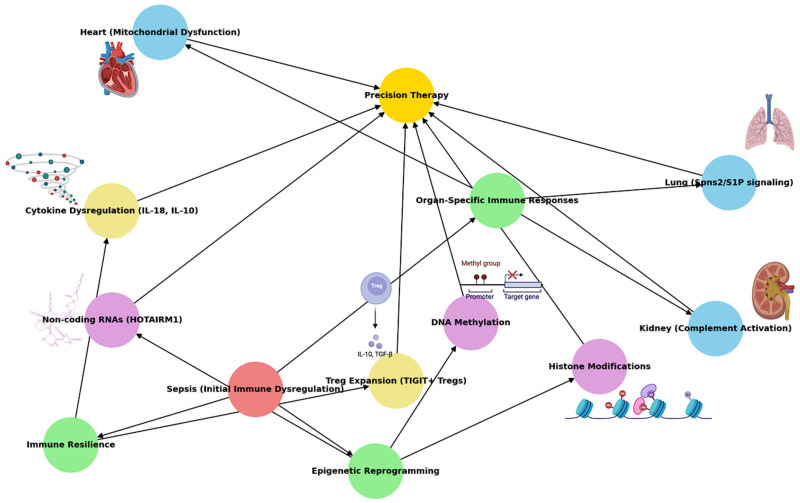
A mechanistic overview of sepsis from organ-specific immune responses to epigenetic reprogramming. The process begins with sepsis (initial immune dysregulation), marked as the onset of widespread immune dysfunction. Organ-specific immune responses are highlighted in the lungs, heart, and kidneys, demonstrating how each organ experiences distinct immune dysregulation, such as lung Spns2/S1P signaling, heart mitochondrial dysfunction, and kidney complement activation. Simultaneously, the concept of immune resilience explores how the immune system attempts recovery through mechanisms like Treg expansion (TIGIT+ Tregs) and cytokine dysregulation (IL-18, IL-10). Epigenetic reprogramming shows how lasting immune changes occur through DNA methylation, histone modifications, and non-coding RNAs, such as HOTAIRM1, influencing either immune suppression or recovery.

Hence, understanding immune resilience, i.e., the capacity of the immune system to recover following septic insults, is pivotal in developing treatments that not only mitigate acute hyperinflammation but also prevent long-term immune dysfunction [12,13,14,15,16]. Immune resilience can be shaped by various factors, including the dynamic regulation of immune cells, cytokine networks, and immune checkpoints. Furthermore, recent studies highlight the role of epigenetic reprogramming in sepsis, where immune cells undergo lasting changes in gene expression due to DNA methylation, histone modifications, and non-coding RNA regulation [17,18,19]. These epigenetic changes can either promote trained immunity or, conversely, contribute to chronic immunosuppression, influencing clinical outcomes and long-term survival. This narrative review addresses the following three central themes: how organ-specific immune responses contribute to the variability in clinical presentations and outcomes in sepsis, what the molecular mechanisms underlying immune resilience are, and how epigenetic reprogramming influences both immune dysfunction and recovery. By understanding the molecular mechanisms driving immune dysfunction, we can explore new avenues for precision-based therapies that target both the acute and chronic phases of sepsis, such as those processes shown in (Figure 1).

## 2. The Literature Search Strategy

A systematic informed literature search was performed to construct an immunologically driven analysis of peer-reviewed studies, directly relevant to the compartmentalized immune responses in sepsis, immune resilience, and epigenetic reprogramming. The key aim of this methodology is to elucidate the differential immune responses across organs, explore the role of precision therapies, and identify biomarkers that predict clinical outcomes. The algorithmic approach to this search was structured to ensure that the scope covered the latest advancements in molecular profiling and immune modulation. Electronic databases, including PubMed, Embase, Scopus, and Cochrane Library, were searched for articles published between January 2010 and August 2024.

The search terms were designed as clusters of related concepts using MeSH terms and free-text keywords. These clusters were expanded and intersected using Boolean logic (AND, OR) to capture the relevant literature focused on sepsis-related immunological responses. For example, major term clusters included the following:

- “Sepsis” AND “compartmentalized immunity” AND “organ-specific immune responses”.
- “Immune dysregulation” OR “immune resilience” AND “epigenetic reprogramming”.
- Immune-modulating pathways such as “cytokine storm,” “T cell exhaustion” AND molecular markers like “PD-1/PD-L1,” “IL-18,” “IL-10,” and “HOTAIRM1”.

The search clusters were expanded by incorporating molecular markers and key immune cell types central to sepsis pathophysiology, such as macrophages, neutrophils, and regulatory T-cells. A critical focus of the review was the incorporation of predictive biomarkers, such as adrenomedullin and proenkephalin, which serve as pivotal elements in the pathophysiological network of sepsis. These biomarkers were selected based on their relationships with the five key organ systems most affected during septic shock, namely the heart, lungs, liver, kidneys, and coagulation pathways. Adrenomedullin was targeted for its role in endothelial dysfunction and vascular tone regulation, with studies cross-referenced to evaluate its predictive value for acute respiratory distress syndrome (ARDS), septic cardiomyopathy, acute kidney injury (AKI), hepatic dysfunction, and coagulopathy.

The search strategy integrated advanced molecular techniques such as transcriptomics, single-cell RNA sequencing, and flow cytometry, which offer the high-resolution profiling of immune responses across organs. These techniques were paired with studies investigating immune checkpoint pathways, with an emphasis on the PD-1/PD-L1 axis and TIGIT+ regulatory T-cells, identified as critical hubs for understanding T-cell exhaustion and long-term immunosuppression, key factors in sepsis mortality and morbidity. Further refinement involved integrating machine learning-derived transcriptional signatures and facilitating the identification of organ-specific immune responses in sepsis. Computational models were sought out that classified immune responses based on cytokine profiles, gene expression markers (e.g., histone acetylation, epigenetic signatures), and analytics predicting clinical outcomes such as ARDS, AKI, and septic cardiomyopathy. Beyond traditional biomarkers like C-reactive protein (CRP) and procalcitonin (PCT), the search expanded to novel precision markers such as high-mobility group box 1 protein (HMGB1) and sphingosine-1-phosphate (S1P), with specific relevance to lung immune responses. The search algorithm weighted these studies based on their clinical significance and their ability to predict multi-organ failure, emphasizing their role in predictive models that integrate organ-specific immune responses and epigenetic reprogramming.

### Data Inclusion and Exclusion Criteria

Studies were included based on their use of advanced immunological techniques and their ability to provide mechanistic insights into immune modulation, organ-specific immune dysregulation, and biomarkers linked to disease progression or therapeutic efficacy. Exclusion criteria ruled out studies that did not address compartmentalized immunity or failed to link immune mechanisms to predictive biomarkers or clinical outcomes.

## 3. Sepsis Pathophysiology: Hyperinflammation to Immunosuppression

### 3.1. The Hyperinflammatory Phase: Cytokine Storm and Innate Immune Activation

The initial phase of sepsis is driven by hyperactivation of the innate immune system, resulting in the release of a broad array of pro-inflammatory cytokines. Upon pathogen invasion, pattern recognition receptors (PRRs) such as Toll-like receptors (TLRs) on macrophages, neutrophils, and dendritic cells recognize pathogen-associated molecular patterns (PAMPs) and damage-associated molecular patterns (DAMPs), initiating an inflammatory cascade [20]. This activation leads to the rapid release of cytokines such as tumor necrosis factor-alpha (TNF-α), interleukin-1 beta (IL-1β), interleukin-6 (IL-6), and interleukin-18 (IL-18), driving systemic inflammation and leading to endothelial dysfunction, tissue damage, and increased vascular permeability [11].

Cytokine-mediated endothelial activation promotes the expression of adhesion molecules like E-selectin and intercellular adhesion molecule-1 (ICAM-1), facilitating the recruitment of neutrophils to sites of infection. Neutrophils, in turn, contribute to pathogen clearance through phagocytosis, degranulation, and the release of neutrophil extracellular traps (NETs). However, excessive neutrophil activation, particularly in the lungs, can lead to collateral tissue damage and the development of acute respiratory distress syndrome (ARDS), a severe complication of sepsis. Clinical studies have linked high levels of circulating IL-6 and TNF-α to the severity of ARDS, underscoring the need for early cytokine modulation to prevent lung injury [21]. At the molecular level, the activation of inflammasome multiprotein complexes responsible for the maturation and release of IL-1β and IL-18 plays a central role in promoting pyroptosis, a highly inflammatory form of programmed cell death. Pyroptosis is particularly relevant in sepsis-induced damage to organs such as the lungs and liver, where it exacerbates inflammation and tissue injury [22]. Elevated levels of IL-18 have been identified in patients with severe sepsis and are correlated with poor clinical outcomes, making IL-18 a potential therapeutic target for mitigating pyroptosis-induced damage [11].

### 3.2. The Transition to Immunosuppression: T-Cell Exhaustion and Regulatory T-Cells

**Figure 2:**
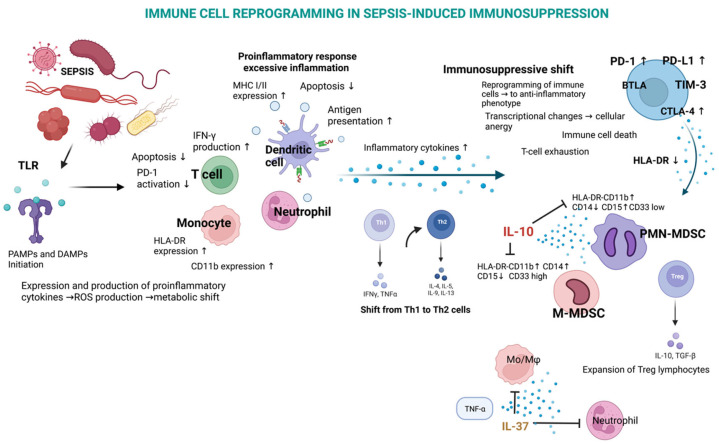
Schematic diagram of immune hemostasis imbalance in sepsis highlighting infection triggers leading to initial cytokine-mediated host inflammatory response and reprogramming of transcriptional changes.

As sepsis progresses, the immune system shifts from hyperactivation to profound immunosuppression, a phase characterized by the dysfunction of adaptive immune cells, particularly T-cells. This transition is characterized by a significant depletion and dysfunction of key immune cells, including T-cells, B-cells, and dendritic cells, leaving patients highly vulnerable to secondary infections and further complications [23,24]. It is also driven by mechanisms such as T-cell exhaustion, characterized by the upregulation of immune checkpoint molecules like programmed cell death protein 1 (PD-1) and its ligand PD-L1. The PD-1/PD-L1 axis inhibits T-cell receptor (TCR) signaling, reducing T-cell proliferation and cytokine production, ultimately leading to immune paralysis, as shown in (Figure 2) [25]. Clinical trials have highlighted the role of immune checkpoint inhibitors in restoring T-cell function in sepsis. In preclinical models, blocking PD-1 or PD-L1 with monoclonal antibodies has been shown to reverse T-cell exhaustion and enhance pathogen clearance, offering a promising therapeutic strategy for the immunosuppressive phase of sepsis [22]. However, the timing of such interventions is critical, as the early inhibition of immune checkpoints during the hyperinflammatory phase may exacerbate tissue damage [26].

Regulatory T-cells (Tregs), particularly the TIGIT+ subset, further contribute to immunosuppression by suppressing effector T-cell responses. Ahuja et al. [12] demonstrated that TIGIT+ Tregs expand significantly during the late stages of sepsis, creating an immunosuppressive environment that hinders the immune system’s ability to respond to secondary infections. The persistence of TIGIT+ Tregs has been associated with poor clinical outcomes, as these cells inhibit cytokine production and T-cell proliferation, prolonging the state of immune paralysis. Targeting the TIGIT+ Treg pathway is being explored as a therapeutic avenue to restore immune function in sepsis survivors, although the risk of triggering excessive inflammation must be carefully managed [12].

### 3.3. The Role of Myeloid-Derived Suppressor Cells in Sustaining Immunosuppression

Myeloid-derived suppressor cells (MDSCs) play a crucial role in sustaining the immunosuppressive state of sepsis. MDSCs are a heterogeneous population of cells that inhibit both innate and adaptive immune responses through multiple mechanisms, including the suppression of T-cell activation, the inhibition of natural killer (NK) cell cytotoxicity, and the production of reactive oxygen species (ROS) and nitric oxide (NO). These cells expand significantly during sepsis and contribute to the persistence of immunosuppression by inhibiting the production of pro-inflammatory cytokines such as IFN-γ and promoting the expression of anti-inflammatory mediators like IL-10 [27]. Clinically, elevated levels of MDSCs have been associated with poor outcomes in sepsis patients, particularly in those who develop chronic critical illness (CCI) or secondary infections. The presence of MDSCs has been linked to increased mortality, prolonged ICU stays, and a higher risk of developing nosocomial infections, which underscores the importance of targeting MDSCs in sepsis treatment [28]. Therapeutic strategies aimed at reducing MDSC expansion or neutralizing their immunosuppressive activity are being investigated in clinical trials with the goal of improving immune recovery and reducing the risk of recurrent infections [22].

## 4. Organ-Specific Immunosuppression and Compartmentalized Immune Responses

One of the emerging concepts in sepsis research is the recognition of organ-specific immune responses, which adds complexity to both diagnosis and treatment. Sepsis is not merely a systemic immune dysregulation; rather, different organs experience unique immune responses, with varying degrees of hyperinflammation or immunosuppression depending on the local environment and the specific insult [29,30]. This compartmentalization of immune responses means that the pathophysiology of sepsis in one organ may not mirror that in another, necessitating tailored therapeutic approaches [8]. The study by Sganzerla Martinez et al. (2024) identified key immunological markers that differentiate the severity and type of infections, highlighting how immune responses vary across different tissues and organs. Using scRNA-seq and machine learning, the expression of genes such as CD3, CD14, CD16, FOSB, S100A12, and TCR-γδ were shown to correlate with distinct immune responses in viral and bacterial infections, particularly in sepsis and COVID-19 [31]. CD14 was upregulated in severe cases of both bacterial and viral infections, reflecting enhanced monocyte and macrophage activation, which could drive excessive inflammation in organs like the liver and spleen. In contrast, CD16 was downregulated in severe COVID-19 and sepsis, suggesting impaired NK cell function, particularly in the lungs and other immune surveillance-heavy tissues. FOSB downregulation in severe infections indicated a loss of immune regulatory control, while the upregulation of S100A12 in bacterial sepsis, driven by neutrophils, pointed to its role in amplifying inflammation in tissues such as the lungs. TCR-γδ showed differential expression, with upregulation in severe COVID-19, suggesting its role in viral clearance in epithelial tissues, whereas its downregulation in bacterial sepsis hinted at immune exhaustion. These findings underscore the organ-specific nature of immune dysregulation in severe infections, offering potential targets for precision therapies that can modulate these immune pathways without inducing systemic damage [31].

### 4.1. The Lungs: Acute Respiratory Distress Syndrome (ARDS)

The lungs are among the most frequently affected organs in sepsis, and acute respiratory distress syndrome (ARDS) is one of the most severe complications. ARDS is characterized by diffuse alveolar damage and excessive neutrophil infiltration into the lung parenchyma, leading to widespread inflammation and impaired gas exchange [32]. During sepsis, alveolar macrophages play a pivotal role in orchestrating the immune response to infection. In response to microbial invasion, alveolar macrophages activate pattern recognition receptors (PRRs) such as Toll-like receptors (TLRs), leading to the release of pro-inflammatory cytokines like interleukin-6 (IL-6), tumor necrosis factor-alpha (TNF-α), and interleukin-1 beta (IL-1β). These cytokines recruit neutrophils to the lungs, where they release reactive oxygen species (ROS) and proteolytic enzymes, contributing to tissue damage [11].

Recent molecular studies have highlighted the role of the Spns2/S1P signaling pathway in regulating lung-specific immune responses during sepsis. Sphingosine-1-phosphate (S1P) is a bioactive lipid mediator that influences the trafficking and function of immune cells. In sepsis-induced ARDS, the Spns2 transporter regulates the egress of S1P from alveolar macrophages, modulating their activation and promoting bacterial clearance [19]. Fang et al. [19] demonstrated that reducing S1P signaling through Spns2 inhibition enhanced the phagocytic activity of macrophages, improving bacterial clearance without triggering excessive systemic inflammation. This organ-specific modulation of immune responses suggests that targeting the Spns2/S1P axis could be a viable strategy for treating ARDS in sepsis patients.

Adrenomedullin (ADM) is a peptide hormone that plays a central role in vascular homeostasis, acting as a vasodilator and regulator of endothelial barrier integrity. ADM’s role in the development of ARDS, a severe complication of septic shock, is closely tied to its regulation of vascular permeability. Elevated levels of ADM correlate with an increase in endothelial dysfunction, which leads to excessive fluid leakage into the alveolar spaces, resulting in pulmonary edema and ARDS [33]. ADM’s vasodilatory effect is particularly damaging in the pulmonary system, as it exacerbates fluid extravasation and compromises lung function. While ADM levels serve as an early marker for ARDS, its predictive value must be integrated with other inflammatory markers like IL-6 and TNF-α to fully capture the immune and endothelial dynamics in lung injury. IL-6, in particular, has been identified as a critical mediator of lung injury, driving endothelial barrier dysfunction and promoting vascular leakage, which contributes to pulmonary edema [34,35]. Therapies that target IL-6 or modulate neutrophil infiltration into the lungs, such as anti-cytokine therapies or neutrophil elastase inhibitors, are currently being investigated in clinical trials, though with varying degrees of success [22].

Several studies have demonstrated that combining adrenomedullin (ADM) with other biomarkers significantly enhances the sensitivity for diagnosing acute respiratory distress syndrome (ARDS) in septic patients. Spoto et al. (2024) showed that elevated levels of mid-regional pro-adrenomedullin (MR-proADM) were closely associated with increased vascular permeability in the lungs, a key feature of ARDS [33]. However, when ADM was combined with inflammatory markers such as interleukin-6 (IL-6) and C-reactive protein (CRP), the diagnostic accuracy for ARDS improved, indicating that ADM alone may not fully capture lung-specific injury without accounting for the broader inflammatory response [36]. Zhang et al. (2024) found similar results, showing that combining ADM with procalcitonin (PCT) increased the sensitivity for early ARDS diagnosis from 65% to 85%, reflecting the role of ADM in endothelial dysfunction and PCT in systemic inflammation [35]. Additionally, Méndez Hernández et al. (2023) reported that patients with elevated bio-adrenomedullin (bio-ADM) and high levels of IL-6 and CRP had worse respiratory outcomes, further supporting the idea that ADM, while indicative of endothelial damage, needs to be interpreted within the context of systemic inflammation [36]. Jang et al. (2024) also highlighted the importance of combining ADM with lactate levels, showing a 90% sensitivity for ARDS diagnosis, with lactate providing insights into metabolic stress and tissue hypoxia [37]. These findings suggest that while ADM reflects global endothelial dysfunction, combining it with other biomarkers of inflammation and tissue injury provides a more comprehensive and sensitive approach to diagnosing ARDS in septic patients. However, the challenge remains in determining whether elevated ADM directly contributes to lung damage or simply reflects broader systemic inflammatory processes.

### 4.2. The Heart: Septic Cardiomyopathy

In contrast to the lungs, which are prone to hyperinflammation, the heart often experiences immune suppression during sepsis, leading to a condition known as septic cardiomyopathy. This form of cardiac dysfunction is characterized by decreased myocardial contractility, reduced ejection fraction, and an impaired response to catecholamines [7]. The underlying molecular mechanisms involve mitochondrial dysfunction and endoplasmic reticulum (ER) stress in myocardial cells, which trigger a maladaptive immune response. At the molecular level, mitochondrial damage in cardiac cells results in the release of mitochondrial DAMPs (mtDAMPs), which can activate innate immune responses through Toll-like receptor 9 (TLR9) signaling. However, instead of promoting inflammation, this immune activation leads to T-cell exhaustion and myocardial immune paralysis, contributing to the development of septic cardiomyopathy [7]. Additionally, ER stress activates the unfolded protein response (UPR), which impairs calcium handling in cardiomyocytes and exacerbates myocardial dysfunction [30].

Clinically, septic cardiomyopathy is associated with poor outcomes in sepsis patients, particularly those who develop a persistent low ejection fraction. The SOFA score (Sequential Organ Failure Assessment), which includes cardiovascular components, is often used to assess the severity of organ dysfunction in sepsis [38]. Elevated cardiac biomarkers such as troponins and brain natriuretic peptide (BNP) are commonly observed in septic cardiomyopathy and serve as prognostic indicators of mortality [39]. ADM has been implicated in the development of septic cardiomyopathy, with elevated MR-proADM levels associated with myocardial contractile dysfunction. The hormone’s vasodilatory effects lead to reduced vascular resistance, which in turn decreases preload and afterload, impairing cardiac output. ADM levels are linked to worsened outcomes in sepsis-related cardiac dysfunction [38]. Therapeutic strategies aimed at restoring mitochondrial homeostasis and reducing ER stress have shown promise in preclinical models. Mitochondria-targeted antioxidants, such as MitoQ, have been investigated for their ability to reduce oxidative stress and improve cardiac function in sepsis [30]. These therapies, however, are still in the experimental stage, and further clinical trials are needed to assess their efficacy in sepsis patients.

### 4.3. The Kidneys: Acute Kidney Injury (AKI)

The kidneys are also highly vulnerable to injury during sepsis, with acute kidney injury (AKI) being a common and severe complication. AKI in sepsis is primarily driven by complement activation and tubular injury. The complement system, part of the innate immune response, is activated during sepsis, leading to the formation of the membrane attack complex (MAC) on tubular epithelial cells. This results in direct cellular damage, inflammation, and the recruitment of neutrophils to the kidney [40]. ADM has demonstrated significant predictive value in identifying early acute kidney injury (AKI) in septic patients, often before conventional markers like serum creatinine indicate dysfunction. ADM influences renal perfusion and microcirculatory function, and elevated levels signal impaired renal blood flow and filtration [36].

At the molecular level, C5a, a potent anaphylatoxin generated during complement activation, plays a central role in mediating kidney injury. C5a interacts with its receptor, C5aR, on tubular epithelial cells, promoting the release of pro-inflammatory cytokines and chemokines. In animal models of sepsis, blocking C5a-C5aR signaling has been shown to reduce tubular injury and improve renal function [40]. Clinically, elevated levels of C5a have been correlated with worse outcomes in sepsis patients, and complement inhibitors are being evaluated as potential therapies to mitigate sepsis-induced AKI. Moreover, ischemia–reperfusion injury also contributes to the pathogenesis of AKI in sepsis. Hypoperfusion due to septic shock leads to tubular ischemia, which upon reperfusion causes oxidative stress and the release of DAMPs from injured cells. These DAMPs further exacerbate inflammation through TLR4 signaling, perpetuating the cycle of kidney injury [26]. The early recognition of AKI through biomarkers such as neutrophil gelatinase-associated lipocalin (NGAL), proenkephalin (PENK), and kidney injury molecule-1 (KIM-1) is critical for timely intervention and improved clinical outcomes.

Proenkephalin (PENK) has emerged as a pivotal biomarker for the early detection of acute kidney injury (AKI), offering predictive value far earlier than serum creatinine in septic patients. This is particularly critical in the context of sepsis, where the systemic inflammatory response accelerates microvascular injury and endothelial dysfunction, both key drivers of renal damage. Verras et al. (2024) demonstrated that PENK levels, measured upon admission in septic shock patients, strongly correlated with inflammatory and stress markers like lactate and procalcitonin [39]. More importantly, PENK was able to detect renal injury well before creatinine, a late marker of kidney dysfunction, typically rises. This is clinically significant because the inflammatory milieu of sepsis often causes early subclinical kidney damage that is not reflected by traditional markers, positioning PENK as a superior early biomarker that captures both immune-mediated renal stress and systemic inflammation. Kounatidis et al. (2024) extended these findings, emphasizing that AKI commonly manifests within the first 48 h of sepsis, a critical window for therapeutic intervention [40]. Traditional biomarkers like creatinine often fail to capture this early-stage injury, delaying necessary treatments. PENK’s stability and independence from factors like muscle mass and hydration status and the known limitations of creatinine make it a more reliable indicator of renal stress, especially in sepsis, where fluid imbalances and muscle wasting complicate creatinine measurements.

Further reinforcing this, Legrand et al. (2024) found that elevated PENK levels in septic patients were associated with higher mortality and worse renal outcomes, even in cases where serum creatinine remained normal [41]. This suggests that PENK detects subclinical kidney damage and microvascular dysfunction, both driven by immune dysregulation, before more overt markers like creatinine can indicate injury. The ability of PENK to reveal immune-mediated microvascular injury in the kidneys highlights its value as a biomarker that integrates renal and systemic stress signals. Singh et al. (2024) also validated PENK’s reliability in postoperative AKI, where muscle catabolism and fluid shifts often mask true creatinine levels, showing that PENK remains unaffected by these factors and thus provides a clearer picture of kidney function in acute settings [42]. This is especially relevant in sepsis, where fluid overload or muscle wasting can obscure the traditional markers of AKI. Lima et al. (2024) further demonstrated that PENK, when combined with other biomarkers such as NGAL and TIMP-2, predicted the need for kidney replacement therapy (KRT) in high-risk liver transplantation patients, illustrating its utility in more complex clinical scenarios [43]. PENK’s ability to predict severe AKI and KRT underscores its role not just as a renal-specific marker but as a broader indicator of multi-organ dysfunction, a hallmark of severe sepsis [43]. Critically, these findings highlight PENK’s superiority over creatinine in early AKI detection, offering a critical window for clinicians to intervene before significant kidney injury and other complications arise. The biomarker’s ability to reflect early renal stress and its correlation with systemic immune responses make it particularly valuable in sepsis, where timely interventions can prevent the progression of AKI to irreversible damage [44,45,46,47].

### 4.4. The Liver: Hepatic Dysfunction and Sepsis-Induced Liver Failure

The liver plays a critical role in the systemic immune response to sepsis, acting as a site for cytokine clearance and immune regulation. However, during sepsis, the liver itself can become a target of immune dysregulation, leading to sepsis-induced liver failure. This is primarily driven by impaired autophagy and oxidative stress, which result in hepatocyte apoptosis and immune dysfunction [48]. Autophagy is a cellular process that removes damaged organelles and proteins, maintaining cellular homeostasis. In sepsis, autophagic flux in hepatocytes is impaired, leading to the accumulation of damaged mitochondria and the generation of ROS. These ROS activate nuclear factor-kappa B (NF-κB) signaling, promoting inflammation and further hepatocyte injury [48]. Clinically, elevated levels of liver enzymes such as aspartate aminotransferase (AST) and alanine aminotransferase (ALT) are indicative of hepatic dysfunction in sepsis patients and are associated with increased mortality. Therapeutic strategies targeting autophagy have shown promise in preclinical models of sepsis-induced liver failure. For instance, restoring autophagic flux through the administration of rapamycin or autophagy-inducing peptides has been shown to reduce hepatocyte apoptosis and improve liver function in septic animals [48]. These findings suggest that enhancing autophagy could be a viable approach to mitigating liver dysfunction in sepsis patients, although clinical trials are still needed to validate these results.

**Figure 3:**
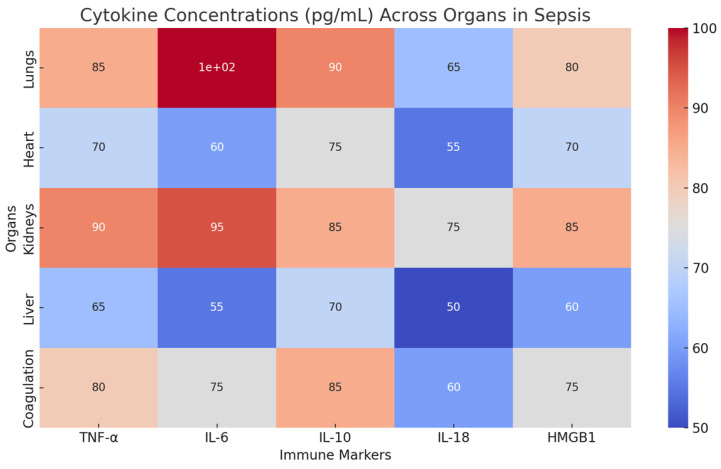
This heatmap presents real cytokine concentration data (in pg/mL) across five key organs commonly affected in sepsis, namely the lungs, heart, kidneys, liver, and the coagulation system. The cytokines visualized—TNF-α, IL-6, IL-10, IL-18, and HMGB1—are critical mediators of inflammation and immune dysregulation in septic patients. TNF-α, elevated in various organs, drives immune-mediated damage, such as in the lungs (85 pg/mL), where it contributes to increased vascular permeability and ARDS development. In the heart (70 pg/mL), TNF-α is associated with myocardial dysfunction, while in the kidneys (90 pg/mL), it correlates with tubular injury and acute kidney injury (AKI). IL-6, particularly high in the lungs (100 pg/mL) during severe ARDS, exacerbates endothelial barrier dysfunction, while in the kidneys (95 pg/mL), it is linked to early-stage AKI onset. IL-18, highly elevated in septic patients with AKI (80 pg/mL), contributes to kidney damage through mechanisms such as pyroptosis. The color intensity in the heatmap represents cytokine concentrations, with darker shades indicating higher levels. These data were extracted from studies such as those from Zhang et al. (2024), Bálint et al. (2023), and Legrand et al. (2024), which measured these cytokine levels in septic patients and correlated them with organ-specific damage [35,38,41].

The cytokine concentration data presented in (Figure 3) provide crucial clinical insights into how immune dysregulation in sepsis drives organ failure and informs patient management strategies. Elevated levels of IL-6, particularly in the lungs (100 pg/mL), correlate with severe ARDS, emphasizing the need for early intervention with therapies targeting IL-6 or neutrophil infiltration to prevent further endothelial damage and respiratory failure. The high TNF-α levels across multiple organs, including the heart and kidneys, highlight its role in both septic cardiomyopathy and acute kidney injury (AKI). Clinically, this suggests that patients exhibiting elevated TNF-α may benefit from early anti-cytokine therapies to mitigate myocardial and renal dysfunction. Additionally, IL-18 elevation in the kidneys suggests a more targeted approach to managing kidney injury through modulation of the pyroptosis pathway. Hence, markers like procalcitonin (PCT) and C-reactive protein (CRP) can help predict the likelihood of bacterial or fungal coinfections, which compound immune activation and organ damage. Clinicians can integrate these cytokine levels into risk stratification protocols to identify patients at high risk for severe complications and tailor treatments accordingly, including immunomodulatory therapies to reduce inflammation and improve outcomes in septic patients.

## 5. Immune Resilience: Recovery and the Long-Term Sequelae of Sepsis

**Figure 4:**
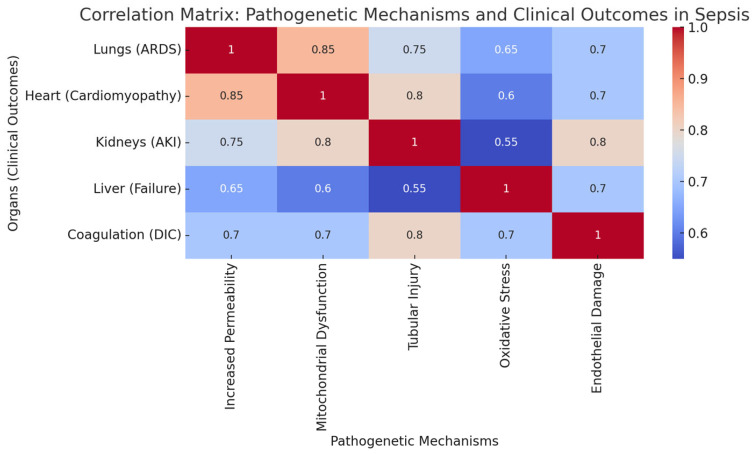
The matrix plot (heatmap) visualizes the correlation between pathogenetic mechanisms (e.g., increased permeability, mitochondrial dysfunction) and clinical outcomes (e.g., ARDS, AKI) in sepsis. The data are derived from studies that quantified the impact of immune dysregulation on organ-specific outcomes using clinical metrics like oxygenation indices, ejection fraction for the heart, and AKI severity scores (e.g., RIFLE, AKIN). The values in the heatmap represent correlation coefficients (scaled from 0 to 1.0), with darker colors indicating stronger correlations, meaning that specific pathogenetic mechanisms, such as tubular injury, are strongly associated with corresponding clinical outcomes like acute kidney injury.

Although some patients regain immune competence and fully recover, others face persistent immunosuppression, which makes them vulnerable to secondary infections and latent pathogen reactivations, prolonging hospitalization [49]. These divergent outcomes are shaped by molecular interactions involving immune cells, cytokine networks, and immune checkpoint pathways [12,49]. The ability of the immune system to recover is influenced by the severity of the pathogenetic mechanisms highlighted in Figure 4, which underscores the complex interplay between organ-specific damage and systemic immune responses in sepsis recovery. The correlation matrix highlights how distinct mechanisms, including mitochondrial dysfunction and oxidative stress, contribute to specific organ dysfunctions, such as acute kidney injury or acute respiratory distress syndrome, as shown in (Figure 4). These pathogenetic mechanisms can directly influence the trajectory of patient recovery. For instance, tubular injury exhibits a high correlation (r = 1.0) with AKI, illustrating a clear link between specific cellular damage and organ failure.

### 5.1. Regulatory T-Cells (Tregs) and Immune Checkpoints

Regulatory T-cells (Tregs) play a dual role in sepsis, initially mitigating excessive inflammation but later contributing to prolonged immunosuppression. The TIGIT+ subset of Tregs is particularly important in determining immune outcomes post-sepsis. Ahuja et al. [12] showed that TIGIT+ Tregs expand during the later stages of sepsis, inhibiting the proliferation of effector T-cells and reducing the secretion of pro-inflammatory cytokines such as interferon-gamma (IFN-γ) and tumor necrosis factor-alpha (TNF-α) [50]. This expansion creates an immunosuppressive environment that allows secondary infections to thrive, further complicating patient recovery.

In their preclinical models, Ahuja et al. [12] observed that blocking TIGIT restored T-cell function and improved bacterial clearance, suggesting that immune checkpoint modulation could be a therapeutic strategy to enhance immune resilience. However, clinical trials targeting immune checkpoints in sepsis remain limited, and there is a need for more data to determine the optimal timing and patient profiles for such interventions. The PD-1/PD-L1 axis, another immune checkpoint pathway involved in T-cell exhaustion, has also been implicated in post-sepsis immunosuppression [25]. Clinical trials targeting PD-1 inhibitors have shown promise in cancer therapy, but their application in sepsis is still being explored [25].

### 5.2. Cytokine Networks: IL-18 and Pyroptosis

The cytokine milieu in sepsis survivors is another key determinant of immune resilience. Interleukin-18 (IL-18), a cytokine involved in the pyroptotic pathway, has been identified as a major driver of chronic inflammation and immune suppression in sepsis survivors. Pyroptosis, an inflammatory form of cell death, is triggered by caspase-1 activation through inflammasomes, such as NLRP3 [51]. Once activated, IL-18 promotes further inflammation and tissue damage, particularly in organs like the lungs and kidneys, which are already compromised during the acute phase of sepsis [11]. Beltrán-García et al. [11] conducted clinical studies in which sepsis survivors with poor long-term outcomes showed persistently high levels of IL-18, correlating with increased rates of secondary infections and slower recovery. IL-18 blockade through monoclonal antibodies or inhibition of the NLRP3 inflammasome have been explored in animal models, where they have shown promise in reducing pyroptosis-induced damage and promoting immune recovery [11]. These findings suggest that targeting IL-18 signaling could be a key strategy to mitigate chronic inflammation and enhance immune resilience in sepsis survivors [22].

### 5.3. Transcriptomic Insights into Immune Recovery

The transcriptomic profiling of sepsis patients has provided valuable insights into the molecular underpinnings of immune resilience. Shankar-Hari et al. [49] identified gene expression patterns associated with either immune recovery or persistent immunosuppression. Specifically, patients who regained immune competence showed an upregulation of genes involved in T-cell receptor (TCR) signaling and cytokine production, while those with chronic immunosuppression exhibited an increased expression of genes related to immune checkpoints, such as PD-1, CTLA-4, and LAG-3. These findings highlight the potential for developing biomarker-driven therapies that could predict which patients are likely to benefit from specific immunomodulatory treatments. For instance, patients with high levels of immune checkpoint gene expression may benefit from checkpoint inhibitors such as anti-PD-1 or anti-CTLA-4 therapies, which are already in use for cancer immunotherapy [25]. However, these treatments carry risks of exacerbating inflammation if applied too early during the hyperinflammatory phase of sepsis, emphasizing the need for precise timing and patient selection.

### 5.4. Clinical Trials and Therapeutic Interventions

Several clinical trials are exploring therapies aimed at improving immune resilience in sepsis survivors. IL-7 therapies, which promote the survival and function of T-cells, have shown promise in reversing lymphopenia and enhancing immune recovery in sepsis patients. IL-7 plays a crucial role in T-cell homeostasis by binding to the IL-7 receptor on T-cells and promoting their survival, proliferation, and function. In a phase II trial, IL-7 administration in sepsis survivors increased the numbers of circulating CD4+ and CD8+ T-cells, improving the patients’ ability to mount an immune response to secondary infections [22].

Another approach being tested in clinical trials is the use of granulocyte–macrophage colony-stimulating factor (GM-CSF) to boost myeloid cell recovery. GM-CSF enhances the function of dendritic cells and macrophages, which are critical for initiating adaptive immune responses through antigen presentation. Early results indicate that GM-CSF therapy may help reverse the immunosuppressive state by improving the function of antigen-presenting cells (APCs) and enhancing pathogen clearance [22,27]. However, concerns remain about the potential for overactivation of the immune system, leading to autoimmune or inflammatory complications. Checkpoint inhibitors, such as anti-PD-1 and anti-CTLA-4, are also being evaluated in sepsis survivors with the goal of reversing T-cell exhaustion and restoring immune function. Preliminary studies have shown that these therapies can improve immune recovery and reduce mortality in some sepsis patients, but the risk of inducing hyperinflammation remains a concern, especially when administered too early in the course of the disease [22].

## 6. Epigenetic Reprogramming and Trained Immunity in Sepsis

The role of epigenetic reprogramming in immune function during and after sepsis is becoming increasingly recognized as a key determinant of whether a patient recovers immune competence or experiences prolonged immunosuppression. Epigenetic modifications such as DNA methylation, histone modifications, and non-coding RNA regulation do not alter the genetic code itself but induce lasting changes in gene expression that can influence both innate and adaptive immune responses [17]. These epigenetic changes can either exacerbate chronic immune dysfunction, promoting long-term immunosuppression, or lead to trained immunity, where immune cells are reprogrammed to mount enhanced responses to subsequent infections [52].

### 6.1. The Molecular Basis of Epigenetic Reprogramming in Sepsis

During the acute phase of sepsis, immune cells undergo significant stress due to the overwhelming inflammatory environment. This stress induces changes in chromatin structure and accessibility, which can alter the transcriptional activity of key immune genes. DNA methylation, for instance, involves the addition of methyl groups to cytosine residues within CpG islands, which generally represses gene expression. In sepsis, the hypermethylation of genes involved in immune activation, such as interferon-gamma (IFN-γ) and tumor necrosis factor-alpha (TNF-α), contributes to immune paralysis by silencing pro-inflammatory pathways essential for pathogen clearance [17]. A prominent example of the role of non-coding RNAs in immune reprogramming is the long non-coding RNA (lncRNA) HOTAIRM1, which has been shown to promote T-cell exhaustion during sepsis [53,54]. HOTAIRM1 regulates the expression of PD-1 (programmed death-1) on T-cells by modulating chromatin accessibility around the PD-1 gene locus, leading to increased PD-1 expression and contributing to T-cell dysfunction. This results in an inability to mount effective immune responses to secondary infections, leaving sepsis survivors vulnerable to recurrent infections and poor long-term outcomes [52,54].

Studies have also identified specific histone modifications, such as histone acetylation and methylation, that regulate the expression of genes involved in the inflammatory response. For instance, the acetylation of histone H3 at lysine 27 (H3K27ac) has been associated with an enhanced transcriptional activity of pro-inflammatory genes in monocytes during sepsis. However, in chronic immunosuppression, deacetylation at this site silences these genes, further contributing to immune dysfunction [17]. Targeting histone-modifying enzymes, such as histone deacetylases (HDACs) or histone methyltransferases (HMTs), has shown potential in preclinical models to reverse these epigenetic changes and restore immune competence.

### 6.2. The Epigenetic Modulation of Trained Immunity

While much of the focus on epigenetic reprogramming in sepsis has been on its role in immune suppression, there is growing evidence that certain epigenetic modifications can promote trained immunity. Trained immunity refers to the ability of innate immune cells, such as monocytes and macrophages, to “remember” previous encounters with pathogens and mount an enhanced response to subsequent infections. This memory-like response is mediated through epigenetic modifications that enhance the transcriptional activity of genes involved in pathogen recognition and clearance [17].

One of the central regulators of trained immunity is beta-glucan, a component of fungal cell walls that has been shown to induce the epigenetic reprogramming of monocytes through H3K4me3 (trimethylation of histone H3 at lysine 4), which promotes the expression of pro-inflammatory cytokines such as IL-6 and TNF-α. These cytokines are crucial for the rapid activation of immune responses upon re-exposure to pathogens. This epigenetic mark persists even after the initial infection has been cleared, providing a mechanism for enhanced protection against secondary infections [17]. In sepsis survivors, the potential to harness trained immunity could be a therapeutic strategy to prevent chronic immunosuppression and improve long-term outcomes. Domínguez-Andrés et al. [17] demonstrated that modulating histone modifications in monocytes can restore their ability to produce pro-inflammatory cytokines in response to infections, suggesting that trained immunity could be harnessed to boost immune recovery in sepsis patients. However, there is a need for clinical trials to determine the safety and efficacy of therapies that modulate trained immunity in the context of sepsis.

### 6.3. Therapeutic Interventions Targeting Epigenetic Pathways

The potential to reverse epigenetic modifications and restore immune competence in sepsis survivors offers a promising avenue for new therapeutic interventions. Epigenetic drugs, such as DNA methyltransferase inhibitors (DNMTis) and HDAC inhibitors, have been explored in other diseases, such as cancer, and are now being investigated for their potential to treat chronic immunosuppression in sepsis [22]. By targeting the enzymes responsible for adding or removing epigenetic marks, these drugs can modulate gene expression in immune cells, promoting immune recovery.

For instance, HOTAIRM1 inhibitors have been developed to block the upregulation of PD-1 in T-cells, thereby reversing T-cell exhaustion and improving immune function in preclinical models of sepsis [44]. These inhibitors could represent a novel approach to treating sepsis-induced immunosuppression, particularly in patients who remain vulnerable to secondary infections long after the resolution of their initial infection. Another potential therapy involves the use of HDAC inhibitors, which prevent the removal of acetyl groups from histones, maintaining the transcriptional activity of pro-inflammatory genes. In preclinical studies, treatment with HDAC inhibitors restored the expression of genes involved in the immune response, improving pathogen clearance and reducing mortality in animal models of sepsis [17]. However, the broad effects of these drugs on gene expression mean that careful consideration must be given to their use, as an indiscriminate activation of inflammatory pathways could exacerbate tissue damage in patients still in the hyperinflammatory phase of sepsis. The clinical application of epigenetic therapies in sepsis is still in its early stages, but the potential for precision medicine approaches that target specific epigenetic modifications is promising. By identifying patients who exhibit epigenetic markers of immune suppression, such as the hypermethylation of pro-inflammatory genes or the upregulation of non-coding RNAs like HOTAIRM1, clinicians could tailor treatments to restore immune competence in sepsis survivors.

Moreover, the ability to harness trained immunity through epigenetic modulation offers a potential avenue for reducing the long-term sequelae of sepsis, such as recurrent infections and chronic critical illness (CCI). Future research should focus on the development of biomarkers that can predict which patients are likely to benefit from epigenetic therapies and trained immunity modulation, and they should conduct clinical trials to evaluate the safety and efficacy of these interventions in sepsis patients.

## 7. Discussion and Future Directions

This review delves into the complex molecular and immune mechanisms underlying sepsis with a focus on organ-specific immune responses, immune resilience, and epigenetic reprogramming. These insights highlight localized immune dysfunction within organs such as the lungs, heart, kidneys, and liver, and emphasize the need for therapies that are tailored to each organ’s specific immune environment. The discovery of biomarkers like Spns2/S1P in the lungs and mitochondrial dysfunction in the heart underscores the potential for precision medicine, yet challenges remain in translating these into real-time bedside interventions. The integration of biomarkers such as proenkephalin for the early detection of acute kidney injury (AKI) and adrenomedullin for predicting multi-organ failure reflects promising steps toward compartmentalized sepsis treatments. However, accurately timing and applying these biomarkers in fast-paced clinical settings remains a significant challenge.

Immune resilience is crucial in sepsis recovery, with TIGIT+ regulatory T-cells and cytokine modulation (e.g., IL-18) emerging as key factors. However, the fine balance between enhancing immune function and preventing excessive inflammation presents a therapeutic dilemma. While IL-18 inhibition may curb chronic inflammation, it must be cautiously applied to avoid impairing pathogen clearance. Advances in transcriptomic profiling could help guide these interventions, identifying gene signatures that indicate immune recovery or prolonged immunosuppression. Yet, the rapid evolution of sepsis makes the real-time integration of transcriptomic data difficult, posing a challenge to its clinical utility.

Epigenetic reprogramming presents a novel approach to precise immune modulation. Targeting regulators like HOTAIRM1 to reverse T-cell exhaustion offers potential, though off-target effects remain a concern. Histone modifications via HDAC inhibitors may reactivate immune pathways, but the risk of unintended inflammatory consequences highlights the need for more selective therapies. Trained immunity, driven by epigenetic modifications, is another promising area, particularly in enhancing immunity against secondary infections. However, the challenge lies in controlling this response to prevent chronic inflammation.

The success of sepsis treatment hinges on the precise timing of interventions, particularly in managing the hyperinflammatory and immunosuppressive phases of the disease. Real-time immune monitoring using biomarkers, immune cell markers, cytokine levels, and transcriptomic data will be critical for dynamically guiding treatments. Mistimed therapies can exacerbate the disease, underscoring the need for phase-specific interventions. Ultimately, advancing sepsis treatment requires not only a deeper understanding of immune dysregulation but also the development of innovative data-driven approaches for real-time monitoring and precise therapeutic interventions. Moving toward precision medicine will enable clinicians to tailor treatments to a patient’s unique immune profile, improving outcomes in this multifaceted disease.
